# Direct monitoring of opto-mechanical switching of self-assembled monolayer films containing the azobenzene group

**DOI:** 10.3762/bjnano.2.93

**Published:** 2011-12-20

**Authors:** Einat Tirosh, Enrico Benassi, Silvio Pipolo, Marcel Mayor, Michal Valášek, Veronica Frydman, Stefano Corni, Sidney R Cohen

**Affiliations:** 1 Weizmann Institute of Science, Department of Materials and Interfaces, Rehovot Israel; 2 Center S3, CNR Institute of Nanoscience, Modena, Italy; 3 Department of Physics, University of Modena and Reggio Emilia, Modena, Italy; 4 Karlsruhe Institute of Technology, Institute of Nanotechnology, PO Box 3640, D-76021, Karlsruhe, Germany; 5 University of Basel, Department of Chemistry, St. Johannsring 19, CH-4056 Basel, Switzerland; 6 Weizmann Institute of Science, Department of Chemical Research Support, Rehovot Israel

**Keywords:** AFM, azobenzene, elastic modulus, molecular dynamics, nanomechanics, photoswitch, quantum mechanics computation, self-assembled monolayer

## Abstract

The potential for manipulation and control inherent in molecule-based motors holds great scientific and technological promise. Molecules containing the azobenzene group have been heavily studied in this context. While the effects of the *cis*–*trans* isomerization of the azo group in such molecules have been examined macroscopically by a number of techniques, modulations of the elastic modulus upon isomerization in self-assembled films were not yet measured directly. Here, we examine the mechanical response upon optical switching of bis[(1,1'-biphenyl)-4-yl]diazene organized in a self-assembled film on Au islands, using atomic force microscopy. Analysis of higher harmonics by means of a torsional harmonic cantilever allowed real-time extraction of mechanical data. Quantitative analysis of elastic modulus maps obtained simultaneously with topographic images show that the modulus of the *cis*-form is approximately twice that of the *trans*-isomer. Quantum mechanical and molecular dynamics studies show good agreement with this experimental result, and indicate that the stiffer response in the *cis*-form comprises contributions both from the individual molecular bonds and from intermolecular interactions in the film. These results demonstrate the power and insights gained from cutting-edge AFM technologies, and advanced computational methods.

## Introduction

Molecule-based motors have great appeal due to their addressability, small size, and the possibility to incorporate them into unique structures. Molecules containing the azobenzene functionality are good candidates for converting light into mechanical work through a facile *cis*↔*trans* isomerization that is controlled by UV and visible light. The forces involved in this transition have been characterized by a number of techniques. For instance, changes in the stiffness of azobenzene-containing films were monitored by nanoindentation [1], by quartz-crystal resonator [2], and by electromechanical spectroscopy [3]. The force exerted per molecule during extension from *cis* to *trans* was extracted from cargo-lifting experiments on a macroscopic Hg droplet [4]. The mechanical response monitored in these works and others like them essentially measures a bulk response, which is governed by several effects including the stiffness of the molecular bond itself, as well as steric effects, electronic coupling, and film structure. Single-molecule force microscopy was used to monitor the mechanical and structural changes in the *cis*↔*trans* transition of individual azo-containing polymer molecules [5–6]. These elegant measurements were simulated by molecular dynamics [7]. It was shown that the mechanical response arises only partly from the azo moiety, and includes contributions from other constituents of the polymer chain.

The ability of azo-containing molecules to self-assemble into monomolecular layers (self-assembled monolayers, SAMs) provides an additional nanometer-scale mechanical system, combining the advantages of single-molecule properties with the coherence and template capabilities of macroscopic structures. These films enable such applications as sensors, and molecular-level mechanical manipulators. As an example, macroscopic transport at the solid–liquid interface was driven by modifying the solid–liquid surface tension at a droplet front by using a molecular switch based on a SAM of rotaxane [8]. Central to the function of such systems are changes in the inter- and intramolecular forces accompanying the transitions. In particular, by virtue of packing into a self-assembled film, steric constraints on the *cis*↔*trans* conversion, which do not exist in the isolated molecule or bulk disordered films, could dominate the switching [9–10]. Strictly, this steric hindrance requires close packing, thus some slight disorder in the film could be an enabling condition for the isomerization [11]. Molecular packing also governs the excitonic coupling between chromophores, which can strongly influence the conversion efficiency [12]. A variety of methods to monitor the *cis*↔*trans* switching have been demonstrated for SAMs. These include mechanical testing, as mentioned above, as well as changes in the local surface potential [13–14], UV–vis spectroscopy [10], wettability [15], and direct molecular-resolution imaging by scanning tunneling microscopy [10]. These methods vary in their ability to resolve the pattern of switching. For instance molecularly resolved images identified concerted switching in a small monolayer domain. And whereas concerted switching in such small domains may provide a path to overcome steric constraints, the fine mechanics of the *cis*↔*trans* conversion in SAMs of azobenzene-containing molecules is still not well understood.

The elastic modulus is a fundamental property based on microscopic properties of the system. As such, it provides a good metric for the isomerization, and is amenable to theoretical computation. Here, we report results of an atomic force microscopy (AFM) and atomistic computational study of the change in local stiffness, as induced by the optical *cis↔trans* conversion in a SAM of 4'-{[(1,1'-biphenyl)-4-yl]diazenyl}-(1,1'-biphenyl)-4-thiol (thio-2-DA). The experimental variation in stiffness shows quantitative agreement with the calculated values.

## Results and Discussion

### Experimental measurements

Measurement of the mechanical properties of monolayer films represents a technological challenge. Nanoindentation is appropriate for direct determination of local stiffness since the measurement is direct and, to first order, model-independent: A local deformation is induced and detected while a calibrated force is applied. Converting the stiffness thus measured to elastic modulus does, however, require a suitable model for the interaction. In this work the Derjaguin–Müller–Toporov (DMT) model was applied, which is appropriate for organic monolayer systems [16]. Another consideration for nanoindentation measurements is the substrate effect. "Buckle's rule" maintains that in order to gain information on the film only, and not the substrate, the depth of penetration into the film must not exceed 10% of the total film thickness. However, this range can be significantly extended in the case of sharp AFM tips [17], and, for soft films on hard substrates, as much as half of the film thickness can be penetrated without experiencing appreciable substrate effects [18]. In any case, film deformation must be kept to a minimum and reliable referencing to the substrate must be made.

**Figure 1:**
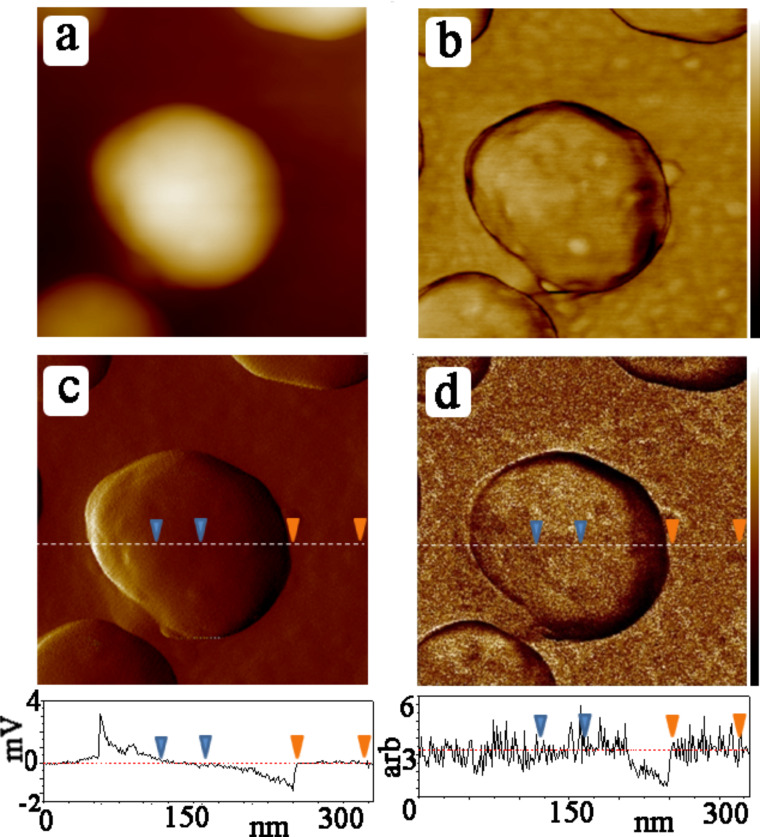
(a) Topography (color bar: 0–70 nm), (b) phase (color bar: 0–15 degrees), (c) error signal (scale indicated in profile), and (d) modulus (scale indicated in profile) of bare Au islands on glass. Cross sections are taken at the same scan line for the error and modulus signals and the triangles demark regions of zero error signal where the modulus measurement is valid (see text). Here, the modulus of the Au islands is the same as that of the glass substrate.

**Figure 2:**
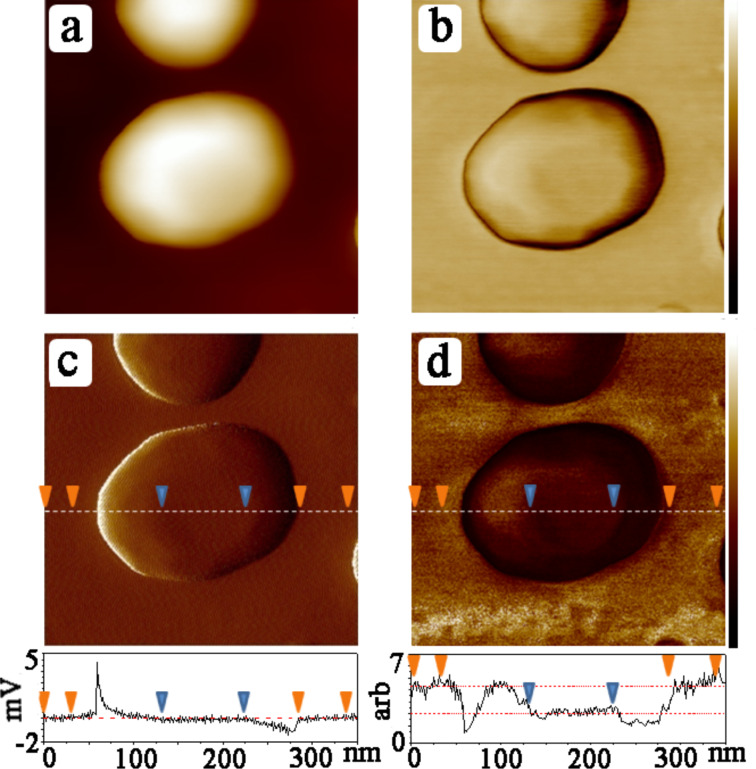
(a) Topography (color bar: 0–70 nm), (b) phase (color bar: 0–15 degrees), (c) error signal (scale indicated in profile), and (d) modulus signal (scale indicated in profile) of SAM-coated Au islands on glass. Data taken from as prepared samples (no irradiation), corresponding to the *trans* configuration.

**Figure 3:**
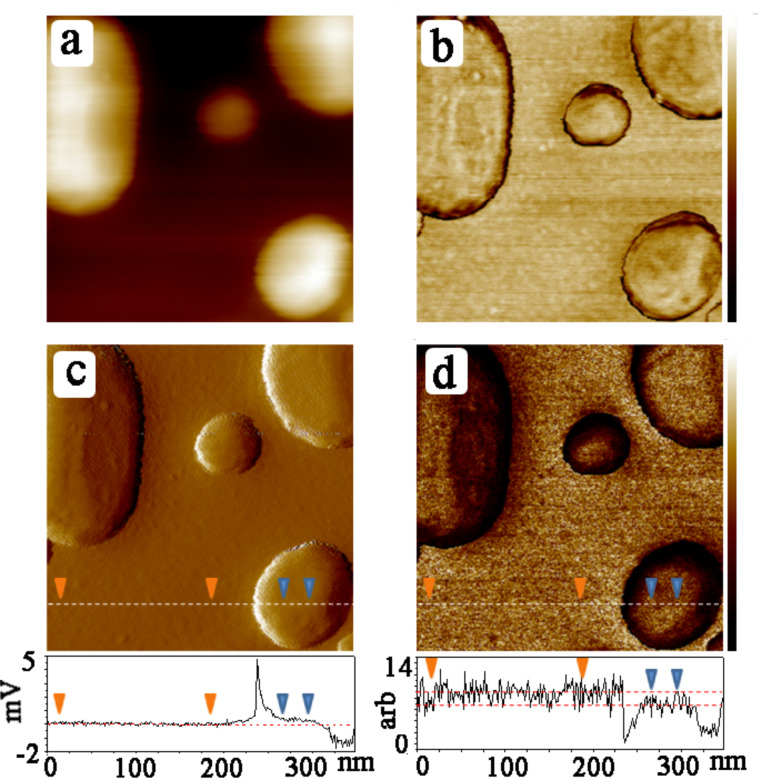
(a) Topography (color bar: 0–15 nm), (b) phase (color bar: 0–20 degrees), (c) error signal (scale indicated in profile), and (d) modulus signal (scale indicated in profile) of SAM-coated Au islands on glass after 120 min of irradiation at 365 nm (see text).

The method applied here is time-resolved tapping force imaging, in which force–deformation curves are reconstructed from the amplitudes of the higher harmonics of oscillation of the flexural mode of the cantilever, spring-coupled to the torsional mode [19]. The latter mode is excited by using a special probe with the tip positioned off of the long axis. Since the force curves are generated simultaneously with the topographic scan, each pixel contains both topographic and mechanical information. Although in principle this method can give absolute modulus values, switching between samples can change probe alignment and hence calibration factors. For this reason, our samples contained an internal standard: The films were formed on Au islands with diameters of several tens of nanometers and a height of 50 nm on a glass substrate. The thio-2-DA molecules bind only to the gold, such that each scan line contains regions of hard surface (glass) and soft surface (SAM/Au). Figures 1–3 show how this concept is used to generate data. For each horizontal scan line, both the glass substrate and the gold island are sampled. For purposes of this measurement, Au and glass are considered equally stiff since the modulus signal saturates at about 5 GPa due to the limits of the cantilever spring constant and the signal sensitivities. The glass surface then serves as an in situ reference to which the film modulus can be compared. Scanning these samples before depositing the SAM gave no modulus contrast between glass and the Au islands, as seen in Figure 1.

Figure 2 shows a measurement in which the Au islands are coated with the SAMs. Images and cross sections show that the film has a significantly lower modulus than the substrate. The modulus is calculated simultaneously with the topography, from the experimentally derived force curves as fit to the DMT equation

**Figure 4:**
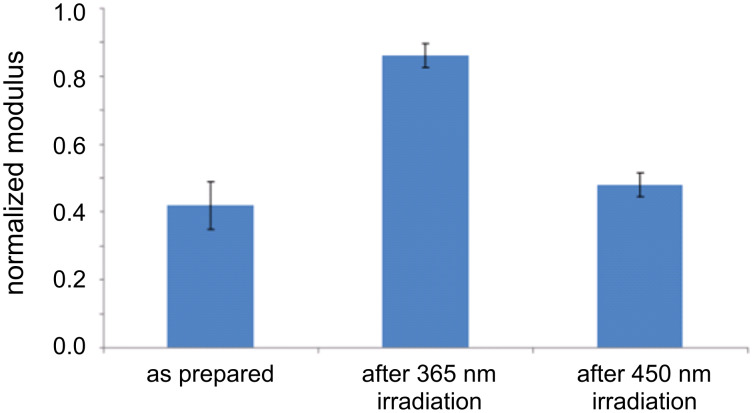
Histogram of the normalized modulus for different illumination conditions: As prepared, at 365 nm for *trans*→*cis* conversion and 450 nm for *cis*→*trans* conversion. The histograms represent collectively analyzed areas of over 80,000 nm^2^, which is the equivalent of over 45,000 pixels of data. The *E_cis_*/*E_trans_* modulus ratio is 1.8 with a relative uncertainty of 20%.

[1]
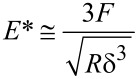


where E* is the reduced modulus, *F* the overall tip–surface force including adhesion, *R* the tip radius and δ the deformation [20]. In principle, individual force curves at specific pixel locations can be stored and analyzed to deduce the local stiffness, but by selecting and averaging entire areas corresponding to the regions of zero error signal as described above, much better statistics were obtained. The main constraint in this case is in the choice of areas of the image where the data can be taken to accurately represent stiffness. This requires monitoring of the corresponding error signal, shown in Figures 1–3. The error signal represents deviation of the modulated tip amplitude from that which is chosen as the feedback setpoint. When this is nonzero, the sample deformation can deviate strongly from the required controlled value. Furthermore, the error signal deviates from zero at the edges of the islands, where the contact area is ill-defined such that *R* in Equation 1 does not provide a good measure of the contact area (the model used here applies to a sphere indenting on a smooth half-plane). For this reason, the topography, error signal, and modulus images were compared to find the proper areas for signal acquisition on the plateau of the islands, with the additional check that the error signal should be less than 0.1% of the total oscillation amplitude. In Figures 1–3 this error value was less than 1 mV out of a 300–500 mV signal. Based on these considerations, the difference in the normalized stiffness of the thio-2-DA SAMs as function of light exposure was measured. Measurements were made on four different samples, with several different tips. Several tens of gold islands were included in the analysis, representing thousands of force–distance curves. The results are displayed in the histogram shown in Figure 4, and in Table 1. The results indicate that the modulus of the *cis*-isomer is approximately twice that of the energetically favored *trans*-isomer.

**Table 1:** Mean values µ, standard deviation σ, relative error, and populations in the statistics for modulus values measured on the different samples.

sample	µ	σ	relative error 100·σ/µ	total area (nm^2^ )	total pixels

Au island	1.02	0.08	8	12760	6830
as prepared	0.42	0.14	33	32070	17160
365 nm	0.86	0.07	8	17830	9540
450 nm	0.048	0.07	15	24490	13100

The illumination conditions were chosen by calibration based on UV–vis spectra of the samples both as solutions and in SAMs. The light sources as described in the methods section were used to illuminate the samples. The thermal back reaction (*cis*→*trans*) was previously verified as being slow in the SAM, with a half-life of 41 min [10]. As prepared, the sample is predominantly in the *trans*-state. By alternately irradiating first at 365 nm and then at 450 nm, the system could be switched between the two states, observed as a reversible transition in the measured stiffness as seen in Figure 4.

### Computational modeling

**Figure 5:**
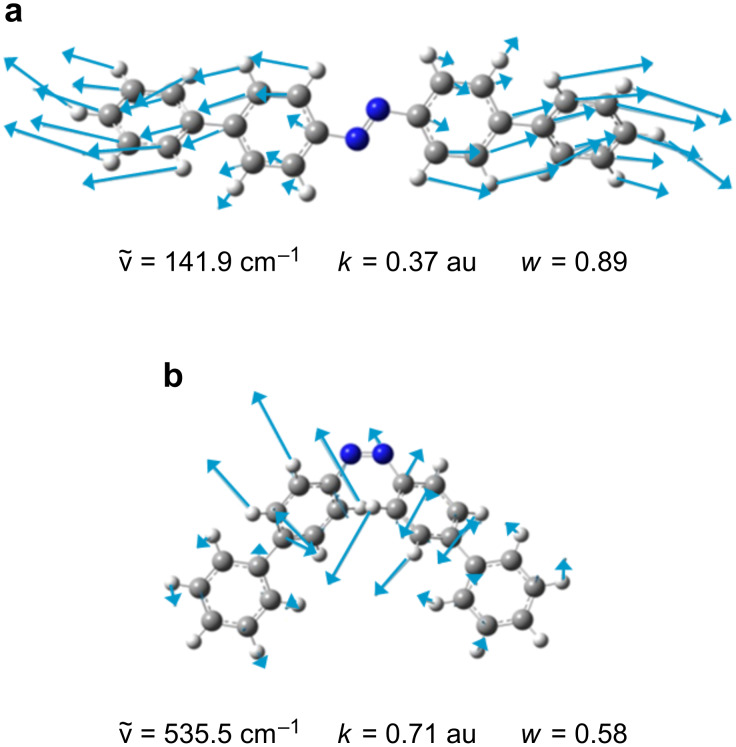
Displacement vectors for the normal mode that dominates the averaged force constant of (a) *trans-* and (b) *cis*-2-DA. The wavenumber (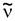), the force constant (*k*, in atomic units, 1 au = 1.56·10^6^ dyn/cm), and the weight (*w*) of each mode within <*k*> are also reported.

The investigation of the relative stiffness of the azobenzene SAM at the molecular level was also approached by computational modeling. The problem was modeled within two different schemes, one based on a quantum mechanical (QM) description of the single molecule, and the other on classical molecular dynamics (MD) simulations of the SAM. In the QM approach, the stiffness of the SAM is first related to a molecular quantity, the weighted molecular force constant <*k*>, through a simple model. Then, <*k*> is obtained by rigorous ab initio calculations (details in Experimental section). The molecular deformations (normal modes) that comprise the major contribution to <*k*> for [(1,1'-biphenyl)-4-yl] [4'-sulfanyl-(1,1'-biphenyl)-4-yl]diazene (2-DA) are shown in Figure 5 for both isomers. For the molecule in the *trans*-configuration, it corresponds to a stretching of the whole molecule along the principle axis. For the molecule in the *cis*-conformation, the dominant normal mode comprises the out-of-plane deformation of the phenyls. The QM model also predicts that the relative *cis*/*trans* stiffness decreases in the series of diphenyldiazene (1-DA) to bis[(1,1'-biphenyl)-4-yl]diazene (2-DA) to bis[(1,1':4',1''-terphenyl)-4-yl]diazene (3-DA), such that the calculated *E_cis_*/*E_trans_* ratios are 2.33, 1.79 and 1.64, respectively. Clearly, the *cis*-configuration is stiffer than the *trans* for all the compounds studied.

**Figure 6:**
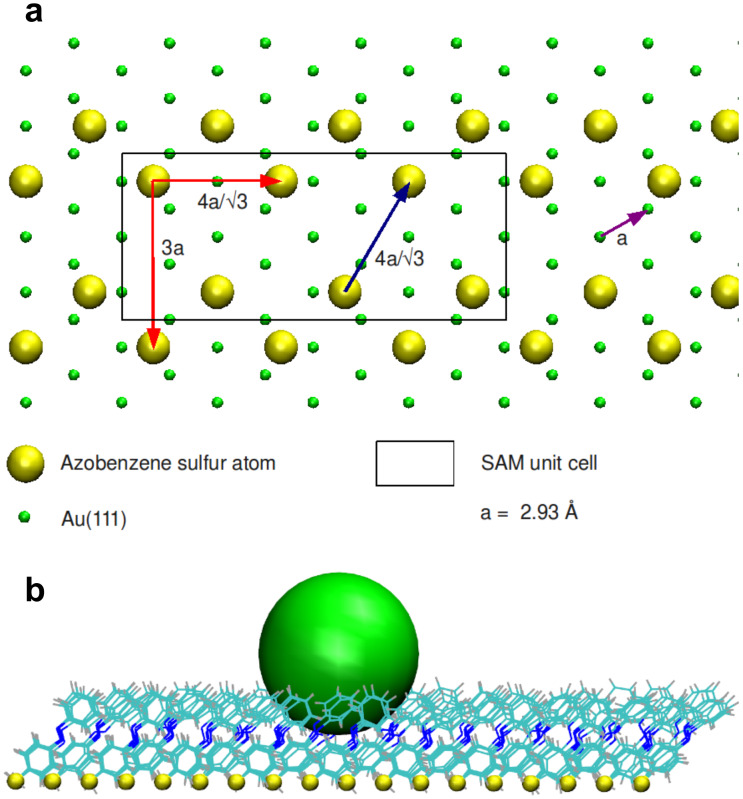
(a) Arrangement of the fixed sulfur atoms in the MD model of the SAM. The unit cell that has been periodically replicated to generate the starting conformation of the SAM is also shown as a black rectangle. It reproduces the periodicity of bright spots in the STM images of [3]. Only the Au atoms of the first surface layer are shown. (b) Snapshot from the MD simulation with the spherical probe (in green) upon the thio-1-DA SAM.

The MD model chosen to mimic the SAM is shown in Figure 6 and is fully described in the Methods section. It uses an atomistic (although empirical) description of the molecules and of their interactions in the SAM, and allows simulation of the compression of the SAM by a nanoindenter. It includes an annealed SAM surface fixed at the base by sulfur atoms, with no explicit inclusion of the gold substrate characteristics. The indenter is an incompressible Lennard–Jones sphere. Whereas the QM model is focused on the single-molecule properties, the MD simulation allows for steric interactions between neighboring molecules.

### Comparison of experiment with computation

As shown in Table 2, the results of the two theoretical approaches are consistent with each other and agree reasonably well with the experimental data. We have also performed test simulations with a MD model that includes the Au surface (described in the Experimental section); the results confirm that the *cis*-isomer is stiffer than the *trans*-isomer also when the surface is included, with a relative stiffness larger than 1 and smaller than 2 for these preliminary calculations. In previous MD simulations of SAMs on gold, it was also found that neglecting the substrate did not qualitatively affect the results [21].

**Table 2:** Comparison of experimental and two different calculated values for the relative stiffness of the *cis*- and *trans*- configurations, *E_cis_*/*E_trans_*.

quantum mechanical^a^	molecular dynamics^b^	experimental

1.8	2.3 ± 0.2	1.8 ± 0.2

^a^Calculated for the 2-DA SAM corresponding to the experiments. The value for 1-DA is 2.33. ^b^Calculated for the thio-1-DA SAM. The uncertainty is the standard deviation from the calculations.

Previous work has generated some questions about the role of steric hindrance in the *cis*↔*trans* conversion within a monolayer film [9–10]. If the film is close-packed, there is some evidence that the conversion is restricted. The specific samples here restrict the domain size to a maximum corresponding to the area on top of the small gold islands, and probably to a much smaller area due to the lack of order induced by the relatively large number of molecules at boundary positions. The calculated values are confined to small systems due to considerations of computational power, but nevertheless may well serve as a good model for the small domains present in the experiment. We have no way to measure directly the efficiency of conversion for the island films. As a comparison, UV–vis spectroscopy performed on smooth, flat, semitransparent Au films, with RMS roughness of 0.7 nm showed only 30% conversion efficiency under similar illumination conditions. We propose that a lower degree of order in SAMs on Au island films allow higher conversion efficiency.

In addition to steric factors, electronic effects such as excitonic and plasmonic coupling have been cited as factors that hinder the switching process. The plasmon spectrum for the Au islands used here peaks at 730 nm, such that any quenching due to the 365 nm irradiation should be a minor effect [22].

The similarity of results from the MD (where intermolecular interactions play the dominant role) and QM (where only single-molecule stiffness is considered) models indicates that the individual molecular bonds and the intermolecular interactions contribute in the same sense to the relative *cis*–*trans* film stiffness. Therefore, it is likely that the higher stiffness of the *cis*-configuration revealed here for partially disordered molecules would hold also for a close-packed SAM of the same molecule, a situation where intra- and intermolecular effects are balanced differently. The QM model seems to be in better agreement with the experiments than the MD one is. This is almost certainly a coincidence, since both models include a number of simplifying approximations. However, based on this observation, one might deduce that for this case accurate modeling of the atomistic properties is more appropriate than inclusion of the overall complexity of the system concomitant with simplifying approximations, contrary to the situation in many cases.

## Conclusion

Relative elastic moduli of the *cis*- and *trans*-isomers of an azobenzene monolayer have been measured and calculated. The modulus ratio of the *cis*- to *trans*-isomer is approximately 2. Results from both the QM-based model (which relates the SAM modulus to the resistance to deformation by individual molecules only) and the MD-based model (which includes intermolecular interactions) agree with this result. Therefore, the *cis*-isomer is stiffer than the *trans*, both as a single molecule and when part of a SAM. Analysis of the individual mode of deformation of the molecule showed that for *trans* there is a predominant normal mode to the stiffness, which corresponds to the molecular stretching/compression along the long axis, which distributes the stress over the entire molecule. For the *cis*-form, the dominant mode represents a deformation sensitive to the stiff steric interactions between the two arms of the azobenzene, and is mainly confined to this local functionality of the molecule (the inner phenyl rings) rather than being delocalized as for *trans*. This provides a microscopic rationale for the observation that the *cis* dominant mode has a force constant larger than the *trans* dominant mode, yielding an overall stiffer molecule.

## Experimental

### Experimental methods

#### Preparation of 4'-{[(1,1'-biphenyl)-4-yl]diazenyl}-(1,1'-biphenyl)-4-thiol (**2**, thio-2-DA)

**Figure 7:**
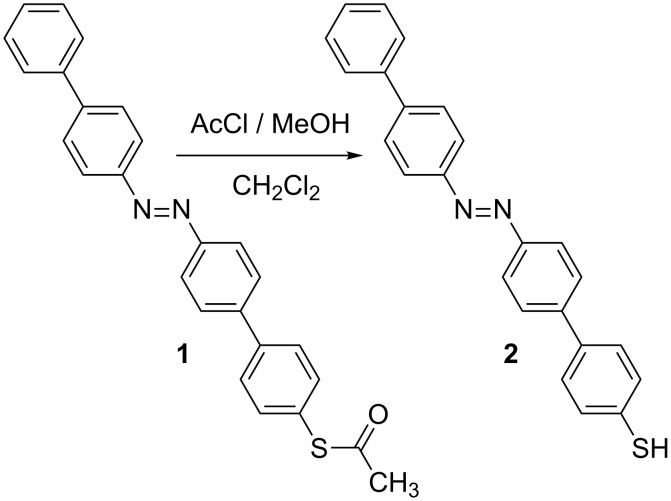
Compound **1** and compound **2** (2-DA-thiol), showing the deprotection reaction yielding the molecule used to form the SAM.

Initial attempts to prepare monolayers directly from compound **1** as reported previously [23–24] were unsuccessful. Therefore, a reduction was carried out as indicated in Figure 7, and outlined below:

Compound **1** (10.1 mg, 0.025 mmol) was suspended in a mixture of deaerated dry CH_2_Cl_2_ (3 mL) and deaerated dry MeOH (2 mL) under nitrogen. The mixture was cooled in an ice–water bath and acetyl chloride (1.4 mL) was added dropwise by a syringe. After the addition was complete, the cooling bath was removed and the mixture was sealed and stirred at room temperature for 4 h. The solvents were then evaporated under reduced pressure affording the thiol **2**, thio-2-DA, which was used without further purification; ^1^H NMR (CDCl_3_) δ 3.5 (s, –S*H*), 7.4 (d, 3H), 7.5 (t, 2H), 7.6 (d, 2H), 7.7–7.8 (m, 6H), 8.0 (m, 4H); ESI–MS (*m*/*z*): [M − 1]^+^ 365.09.

#### Monolayer preparation

**Figure 8:**
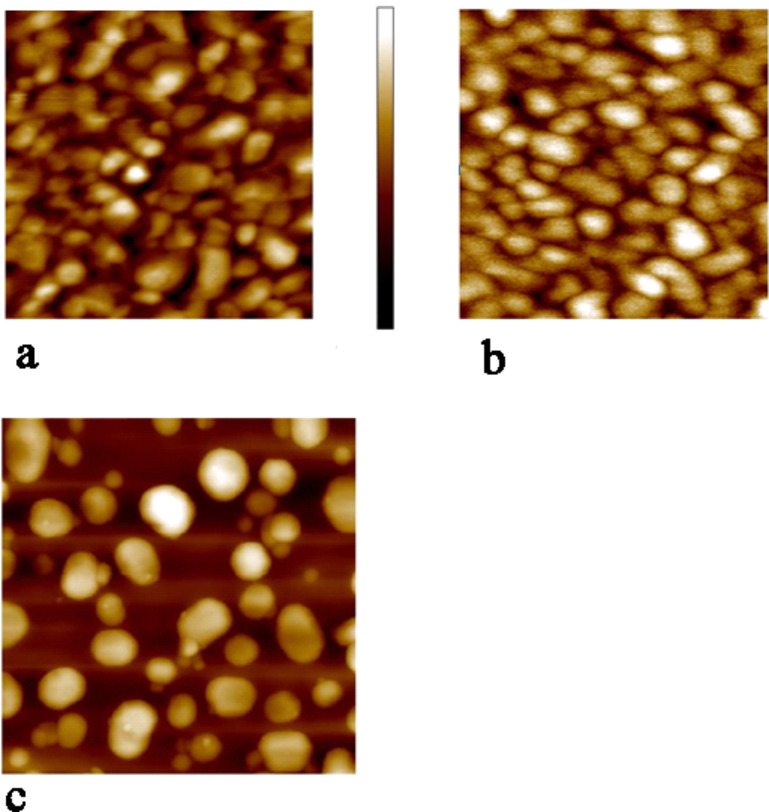
AFM images of (a) clean evaporated Au surface (500 × 500 nm^2^ color bar 12 nm) and (b) surface coated with SAM (500 × 500 nm^2^ color bar 12 nm); (c) Au islands on glass (1300 × 1300 nm^2^ typical island height 50 nm).

**Gold substrate preparation:** AFM images of the different substrates are shown in Figure 8. Three types of gold substrates were used. For basic characterization of the monolayers (ellipsometry, AFM topography, XPS), a 150 nm gold film was prepared on Si by thermal evaporation. For UV–vis measurements, a 20 nm thick Au film was evaporated onto a quartz slide to allow sufficient transmission in the spectral region studied. For the nanomechanical measurements, Au islands on glass substrates were prepared.

**Au island preparation:** 15 nm of Au was evaporated at a deposition rate of 0.01 nm/s onto a clean glass slide. Au islands were developed upon annealing in air at 550 °C for 10 h [22]. The gold island sizes were in the range of 20–150 nm in diameter.

**Au film preparation:** Electron beam deposition from a Au target (99.99%) was performed with a deposition rate of 0.05 nm/s on top of 2 nm of Cr. The Cr serves as an adhesion layer between the gold and the substrate (Si/quartz). Prior to evaporation, the substrates were cleaned by piranha solution for 30 min, followed by copious rinsing with double distilled water (DDW) followed by sonication in ethanol and drying with nitrogen. Substrates for UV–vis analysis were prepared on quartz, with a Au thickness of 20 nm; substrates for other analyses were prepared on Si, with a Au thickness of 150 nm.

**Preparation of monolayer films:** All film preparation, as well as characterization and irradiation experiments were performed at room temperature, 23 ± 1 °C. Before adsorption, substrates were cleaned by a 20 min UV/ozone treatment followed by a 20 min immersion in ethanol. These cleaned Au substrates were immersed in a <0.1 mM solution of thio-2-DA (compound **2** in Figure 7) in degassed dimethylformamide (DMF) at room temperature for 24 h. After adsorption, the samples were rinsed with pure DMF and ethanol and blown dry with nitrogen. The monolayer quality was verified by ellipsometry, X-ray photoelectron spectroscopy, and AFM.

#### Ellipsometry

Ellipsometric measurements were carried out with a variable-angle spectroscopic ellipsometer WVASE32 (J.A. Woollam Co.) with a xenon source and a 1 mm spot at an angle of incidence φ = 70°. The film thickness was calculated by using a Cauchy model for the organic layer. The clean gold substrate was used as a reference. The thicknesses of the samples were in the range of 2.00–2.35 nm, which includes the expected value for the *trans*-SAMs.

#### X-ray photoelectron spectroscopy

XPS spectra were measured on an Axis-Ultra (Kratos, Manchester, UK) system. The characteristic N-peak was clearly seen. Attenuation of the Au signal indicated a film thickness of approximately 2.6 nm. The extent of the coverage was estimated to be close to 100%.

#### Irradiation parameters

**Figure 9:**
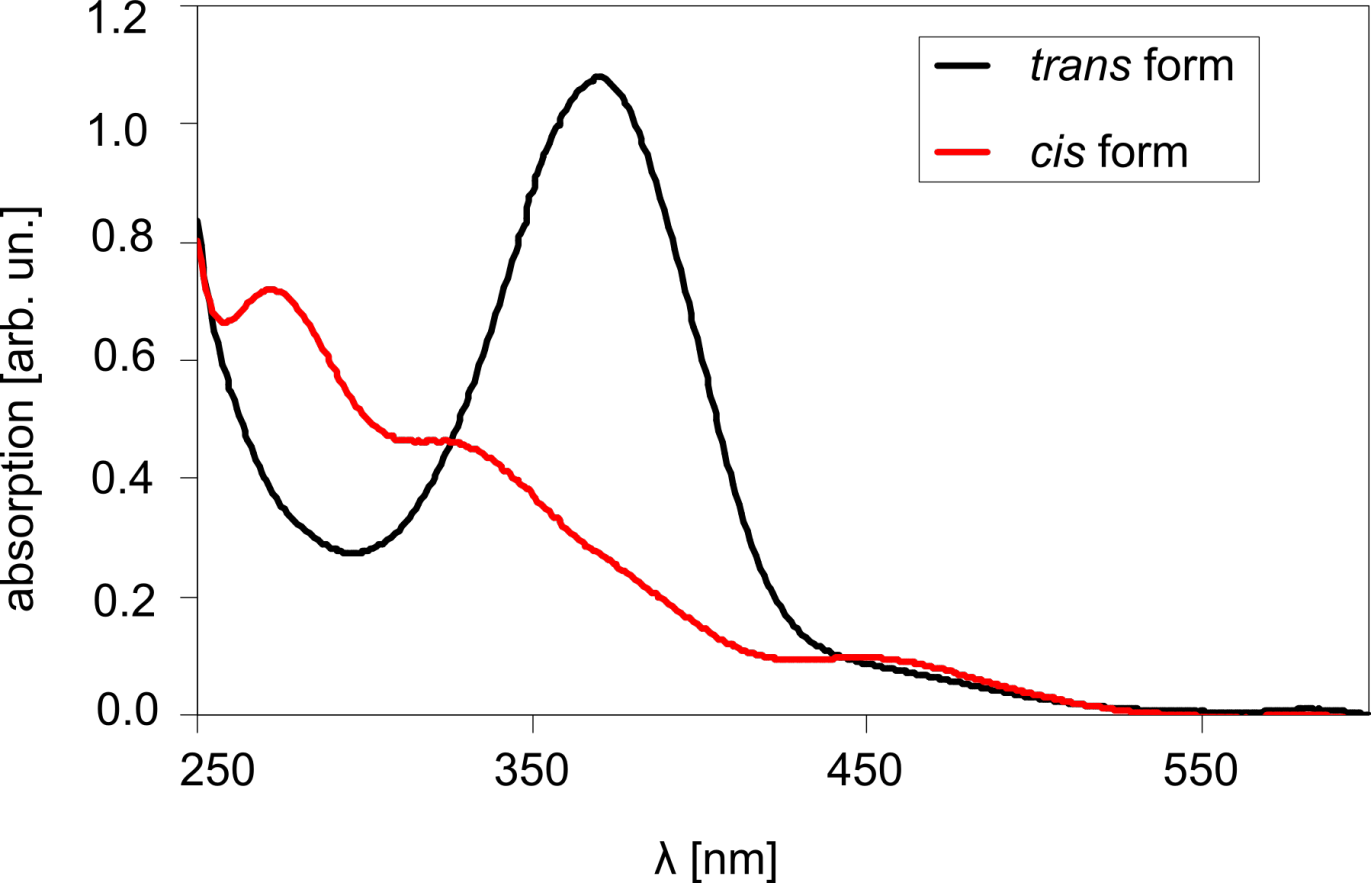
UV–vis spectra for thio-2-DA in chloroform solution after exposure to 365 nm light (*cis* form) and 450 nm light (*trans* form). Arbitrary units indicated in abscissa, since air was used with reference beam. See text for measurement conditions.

The thio-2-DA molecules in solution were irradiated with UV light (wavelength λ = 365 nm; intensity *I* = 25 mW/cm^2^) for up to 20 min. Irradiation of the molecules in solution gave quantitative conversion within 15 min of irradiation (Figure 9).

The azobenzene SAMs were irradiated with UV light (λ = 365 nm, *I* = 25 mW/cm^2^) for 2 h and with visible light (λ = 450 nm, *I* = 5 mW/cm^2^) for 1 h.

#### Scanning probe microscopy

AFM topographies were measured before and after SAM adsorption to check the monolayer quality. Tapping mode AFM measurements were carried out in air with a Multimode Nanoscope V AFM (Veeco, Woodbury, NY). Integrated Si tips (Olympus AC240, resonance frequency ca. 70 kHz) were used for these measurements. Images of the morphology of bare Au and the azobenzene on Au on Si samples are shown in Figure 8. Mechanical characterization was performed in the AFM by using HarmoniX^TM^ imaging (Bruker, Santa Barbara, CA USA). The HarmoniX AFM technique allows the acquisition of quantitative "images" of mechanical parameters (elastic modulus, adhesion, dissipation) simultaneously with and at the rate of acquisition of the tapping-mode image. This is done by analysis of higher harmonics in the oscillating cantilever signal in order to extract full force versus distance curves. A full description of the technique can be found in the literature [19,25]. Since the force curves and stiffness data are derived from the complex probe behavior and require instrumental stability after the necessary calibrations have been performed, the stiffness values reported here are comparisons between different regions, as sampled within a single scan line, which significantly reduces the uncertainty. Preliminary experiments showed no mechanical contrast within the films, except for some dispersed dots that likely represent a contamination on the gold. These dots had lower modulus and adhesion than the surrounding areas.

### Computational methods

#### Quantum mechanical model

**Figure 10:**
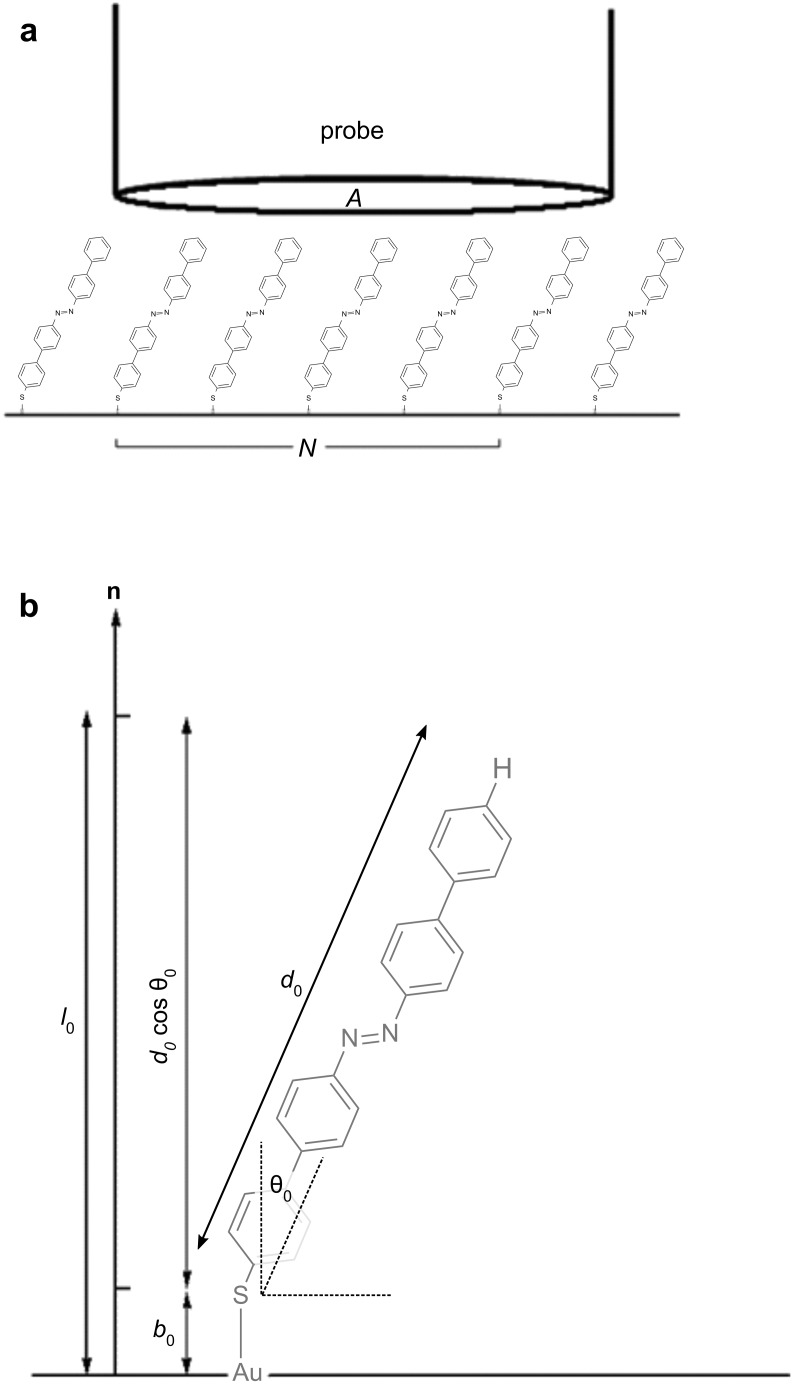
Sketch of the model used to derive SAM stiffness from QM results on the single molecule. (a) *A* is the probe area, *N* is the number of compressed molecules that occupy the area *A*; (b) schematic representation of the geometrical parameters of the QM model: *d*_0_ is the molecule length, *b*_0_ is the S–Au bond length, θ_0_ is the tilt angle, and *l*_0_ is the SAM thickness.

When an area *A* of the SAM is compressed by a force *F* (Figure 10), the SAM thickness changes by Δ*l* = *l*_0_ − *l*, where *l*_0_ is the initial equilibrium thickness and *l* the compressed thickness. If the material is assumed to be homogeneous and isotropic, its Young’s modulus *E* is given by

[2]
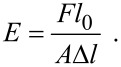


We assume the molecules to behave as ideal (harmonic) springs, homogeneously distributed on the surface. The SAM is thus a collection of parallel springs aligned perpendicular to the surface, each with an elastic (force) constant *k*. Under this assumption:

[3]
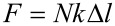


where *N* is the number of molecules that occupy the area *A* (we assume that *N* is the same for *cis*- and *trans-*azo-SAMs). Therefore:

[4]
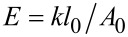


where *A*_0_ = *A*/*N* is the area of gold surface that one single molecule covers, and *l*_0_ is obtained in our model as the projection of the molecular length *d*_0_ (calculated as the largest interatomic distance between sulfur atom and an hydrogen atom) on the normal direction **n** with respect to the gold surface plus the S–Au bond length *b*_0_ (Figure 10b). θ_0_ is the tilt angle for *cis* and for *trans*; this angle was obtained after MD simulations.

From a molecular point of view, the force constant *k* for a deformation perpendicular to the surface can be evaluated from vibrational spectra as a weighted sum over all the normal modes *i*. The weighting is needed to account for the different contributions along the normal direction **n** to the gold plane from the individual normal modes. In order to evaluate this, the force unit vector is decomposed into its Cartesian components *u* in the molecular coordinate reference system, and the weight *w_iu_* is calculated as the product of the component of the normal mode *i* in the direction *u* with the *u-*th component of the force unit vector:

[5]
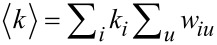


Each *k_i_* is related to the vibrational angular frequency ω*_i_* and the reduced mass μ*_I_* computed after the force matrix diagonalization:

[6]
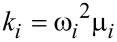


These vibrational frequencies, reduced masses and normal modes were obtained by ab initio QM calculations. A full geometry optimization of the electronic ground state of 1-DA, 2-DA, and 3-DA, both *trans*- and *cis*-isomers, was obtained in the vacuum phase at the level of density functional theory (DFT) by using the Becke three-parameter Lee–Yang–Parr (B3LYP) exchange–correlation functional with cc-pVTZ basis set. The optimized geometries were then subject to vibration calculation in order to compute the vibrational properties and to investigate whether the convergence points were genuine energy minima. For all the calculations, the Gaussian 09 computational package was used [26].

In this framework, after QM computation, we obtained the relative structure factors *l*_0,_*_cis_*/*l*_0,_*_trans_* = 1.061, 0.872, and 0.677 and the ratios between the average force constants <*k_cis_*>/<*k_trans_*> = 2.196, 2.053, and 2.422, for 1-, 2-, and 3-DA respectively. From these values, the *E_cis_*/*E_trans_* values reported in the main text are recovered.

Finally, we also performed a test to evaluate the role of the Au–S–azobenzene bending angle in determining the stiffness ratio. In fact, in our QM model this bending is neglected. We therefore computed vibrational frequencies and normal modes for a thiolated azobenzene (thio-1-DA) molecule, where we gave to the H atom of the thiol group the atomic mass of gold. From such vibrational data we computed again the ratio <*k_cis_*>/<*k_trans_*>, finding a negligible (<1%) difference with respect to the data previously obtained. This is due to the upright orientation of the molecules in the SAM, which makes the bending unable to absorb the external compression.

#### Molecular dynamics approach

The problem of calculating the relative stiffness can also be treated through a classical molecular dynamics approach. An OPLS-type empirical force field [7] is combined with standard OPLS parameters [27] in order to describe the intra- and intermolecular interactions of the SAM. Point charges are derived from the electrostatic potential (RESP) calculated at a B3LYP/cc-pVTZ level of theory on the *trans*-thio-2-DA geometry.

The structure of the SAM was built to reproduce the experimentally observed periodicity [10] (Figure 6) and the gold surface is described, in this first model, only implicitly by fixing the sulfur atom positions. Molecular dynamics (MD) simulations within the canonical ensemble at *T* = 300 K were run considering 126 thio-1-DA molecules in a 6.090 nm × 6.153 nm simulation supercell, with periodic boundary conditions applied. We apply periodic boundary conditions also in the direction perpendicular to the surface (the box size is 7.074 nm along this direction), allowing effective calculation of the electrostatic forces. The Nose–Hoover thermostat [28] was used (time constant for coupling of 0.1 ps). The time step for the simulations was 2 fs (bond lengths were constrained with the LINCS algorithm) [29]. The long-range electrostatic contribution was computed with the PME method with a direct-space cutoff of 1.2 nm. For van der Waals interactions, a switch cutoff of 1.0–1.1 nm was used.

In order to simulate the compression experiments, a computational protocol was set up: First a simulation was run with a spherical indenter positioned at a certain, fixed distance from the plane of the sulfur atoms (Figure 6b). The system was equilibrated for 2 ns, then, with the simulation still running, forces acting on the indenter were collected in the ensuing 8 ns. At the end of this simulation the distance between the indenter and the plane of the sulfur atoms was lowered, and a new (2 + 8) ns simulation was started. We considered 10 different indenter–surface distances. Therefore, a total of 100 ns of MD were run for each compression. This procedure was applied to both *trans* and *cis* thio-1-DA SAMs, and four independent compressions (consisting of 10 simulations each) were run for each isomer, for a total of (2 × 4 × 10 × 10) ns = 800 ns of MD simulations. The four independent compressions were started by four snapshots of equilibrated MD simulations (5 ns long) for the noncompressed *cis*- and *trans*-SAMs, chosen every 1 ns.

**Figure 11:**
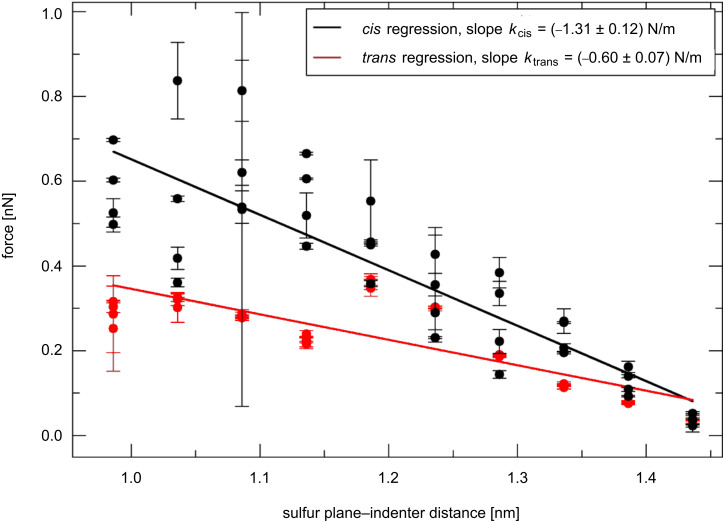
Computational compression procedure: Force acting on the indenter as a function of the distance between the indenter and the plane of the sulfur atoms. Each point refers to a step of the simulation sequence. Error bars are ±σ, where σ is the statistical deviation obtained for each simulation by statistical block average analysis.

By block averaging [30] the forces collected for each simulation, we construct a force–distance plot (Figure 11). The error bars reported in Figure 11 represent the standard deviation from the mean, as estimated by the block-averaging technique for each simulation. They may be unrealistically small when the system remains trapped in metastable states. We minimized this problem by repeating the compressions four times, starting from four different initial conditions, and averaging the results. The ratio of the elastic moduli *E_cis_*/*E_trans_* is calculated considering a thickness ratio *l*_0,_*_cis_*/ *l*_0,_*_trans_* equal to 1.054 (estimated from simulations without the indenter). The indenter is a Lennard–Jones sphere with parameters set as: ε = 0.065 kJ/mol and σ = 1.425 nm. ε is chosen to give a negligible attraction with the SAM (it is one-tenth of the ε used in the GolP model [31] for Au atoms), and σ gives a van der Waals radius of 0.8 nm for the indenter, which is compatible with our cell size. The van der Waals interactions between the indenter and the surface are neglected in this model. As the Au substrate is missing, the SAM–substrate van der Waals interactions are also neglected. Both of these interactions would affect the *cis* and the *trans* force–distance plots in the same way, so their effects on the *E_cis_*/*E_trans_* ratio should be small.

To check the possible role of the gold surface, including the SAM–substrate van der Waals interactions, we also performed test calculations with a second model, where the gold surface was explicitly considered by employing the GolP model [31]. Azobenzene was described with the same OPLS-type parameters mentioned above, with additional literature parameters for the gold–sulfur bond [32]. A computational protocol for the SAM compression similar to that described above was applied within this second model; the simulated system size and the procedural settings were the same as the previous protocol, except that two series of simulations were run for each isomer (instead of four), and the MD simulation for each distance was shorter (5 ns instead of 10 ns). Furthermore, the reference distance for penetration was calculated between the indenter centre and the plane of the surface gold atoms (as sulfur atoms are not fixed). As described in the main text, the results of these tests were qualitatively similar to those of the model that did not explicitly include the Au substrate. While these simulations are valuable as tests to approximately estimate the role of Au, in particular of the Au–SAM van der Waals interaction, further work is needed to properly assess the choices specific to these simulations, such as the Au–SAM force field, the arrangement of the SAM with respect to the Au lattice and the role of Au mobility. All simulations were carried out with the GROMACS package [33].
